# Multiferroic, Phonon and Optical Properties of Pure and Ion-Doped YFeO_3_ Nanoparticles

**DOI:** 10.3390/nano11102731

**Published:** 2021-10-15

**Authors:** Angel Apostolov, Iliana Apostolova, Julia Wesselinowa

**Affiliations:** 1 Civil Engineering and Geodesy, Hristo Smirnenski Blvd. 1, University of Architecture, 1046 Sofia, Bulgaria; angelapos@abv.bg; 2 Kl. Ohridsky Blvd. 10, University of Forestry, 1756 Sofia, Bulgaria; inaapos@abv.bg; 3 J. Bouchier Blvd. 5, Sofia University “St. Kliment Ohridski”, 1164 Sofia, Bulgaria

**Keywords:** ion-doped YFeO3 nanoparticles, magnetization, polarization, phonon energy, band gap, microscopic model

## Abstract

The magnetic, electric, phonon and optical properties of pure and ion-doped orthorhombic YFeO${}_{3}$ nanoparticles are studied for the first time theoretically. The spontaneous magnetization $M_{s}$ in YFeO${}_{3}$ decreases with decreasing particle size. $M_{s}$ is also shape dependent. The magnetization increases by Co and Er ion doping and decreases by Ti doping, which is caused by the different strain which appears in the nanoparticles and changes the exchange interaction constants in the doped states. The phonon energy for the $A_{g}$ mode ω = 149 cm${}^{-1}$ and their damping decreases or increases with increasing temperature, respectively. Both show a kink near the Neel temperature, $T_{N}$, which disappears by applying an external magnetic field. The influence of different ion doping on the band gap energy is also discussed. The doping effects can be used for different applications.

## 1. Introduction

Yttrium orthoferrite YFeO${}_{3}$ (YFO) is of interest due to its significant physical properties connected with different micro-technological devices [1,2,3,4,5]. YFO is an antiferromagnet with a super-exchange magnetic interaction between two Fe${}^{3+}$ ions, the arrangement of which is not perfectly antiparallel, leading to a weak ferromagnetism with a Neel temperature of $T_{N}$∼640 K [1,2]. Moreover, a saturation polarization at room temperature is observed [2,5,6,7], which is an evidence for the multiferroism of YFO. YFO is a type II multiferroic. The microscopic origin of the ferroelectric polarization is considered a spin-exchange striction [7]. The Raman spectra of YFO have been investigated by Raut et al. [8], Saha et al. [9] and Coutinho et al. [10]. Raut et al. [8] have reported an anomalous phonon behaviour near $T_{N}$.

The doping of YFO with different ions—magnetic or nonmagnetic—can improve its magnetic, electric and dielectric properties. Many authors have studied the changes in the properties of YFO which occur though the substitution of different ions on Y-, Fe- or both sites in YFO bulk and nanoparticles [11,12,13,14,15,16,17,18,19,20,21,22,23,24,25,26]. The doping ions have different ionic radii in comparison with the host ions, which leads to strains and to modification of the properties after ion doping. Let us emphasize that there are some discrepancies in the reported results, for example, by Ti ion doping; see Refs. [14,15,16]. We will try to clarify them.

There are not so many theoretical papers which have investigated the multiferroic, phonon and optical properties of doped YFO, either in bulk and nanoparticles. Usually, the magnetic properties of the undoped bulk compounds are considered. The magnetic interactions in *R*FeO${}_{3}$, with *R* = yttrium or a rare earth, have been reported already by Treves [27]. In order to explain the low-energy magnetic excitations of YFO and LaFeO${}_{3}$, Park et al. [28] have used a spin Hamiltonian taking into account the Dzyaloshinsky–Moriya interaction (DMI). The electronic structure and the magnetic properties of the YFO perovskite have been studied by Stoeffler and Chaker [29] using the density-functional theory with the so-called Hubbard correction. Using a first-principles study, the structural, ferroelectric and optical properties of pure and Bi-doped YFO were analyzed recently by Martinez-Aguilar et al. [30].

In the present work, using a microscopic model and the Green’s function technique, we will investigate the size and ion doping effects on the multiferroic, phonon and optical properties of orthorhombic YFO bulk and nanoparticles.

## 2. Model and Methods

The multiferroic properties of YFO are described by the following Hamiltonian:

(1) $$H=H_{m}+H_{e}+H_{me}.$$

The first term in Equation (1) is a modified Heisenberg’s Hamiltonian for the magnetic behavior:

(2) $$\begin{array}{ccc} H_{m} & = & -\sum_{ij}\left(1-x\right)J_{ij}^{Fe-Fe}S_{i}^{Fe}\cdot S_{j}^{Fe}-\sum_{ij}xJ_{ij}^{Fe-DI}S_{i}^{Fe}\cdot S_{j}^{DI}\\ & - & \sum_{<ij>}J_{il}^{{}^{\prime }Fe-Fe}S_{i}^{Fe}\cdot S_{l}^{Fe}-\sum_{ij}D_{ij}\cdot \left[S_{i}^{Fe}\times S_{j}^{Fe}\right]-K\sum_{i}{\left(S_{i}^{zFe}\right)}^{2}\\ & - & g\mu_{B}\sum_{i}h\cdot S_{i}^{Fe}, \end{array}$$

where $S_{i}$ is the Heisenberg spin operator of the Fe${}^{3+}$ ion, and $J_{ij}$ and $J_{il}^{\prime }$ are the exchange interactions between the nearest neighbours and next-nearest neighbours. $J^{Fe-DI}$ is the exchange interaction between the Fe and the doping ions (DI). $D_{ij}$ represents the DMI vector. *K* is the single-ion anisotropy. h is an external magnetic field. *x* is the concentration of the doped ions at Fe states.

**Figure 1:**
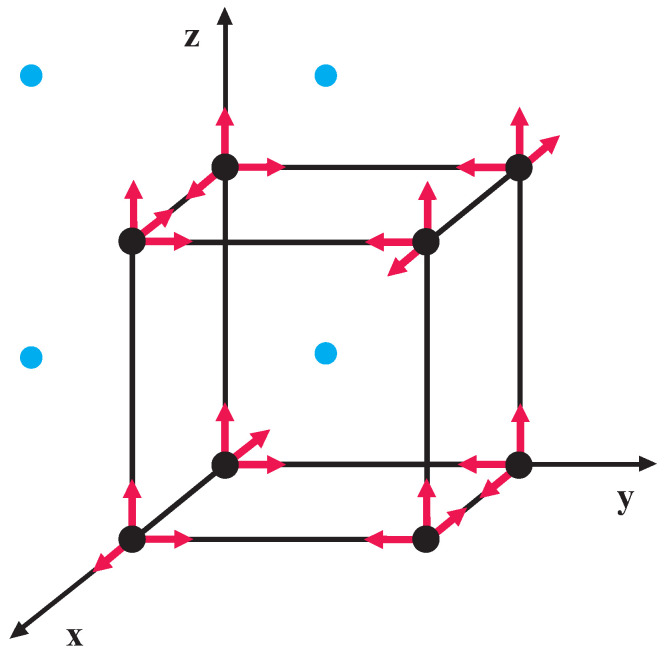
(Color online) Schematic presentation of the directions of the components of the Fe${}^{3+}$ spins (black circle) and the position of the non-magnetic Y ions (blue circle) in the magnetic phase.

In Figure 1, a schematic presentation is given of the directions of the components of the Fe ions (open circle) and the position of the non-magnetic Y ions (full circle) in the magnetic phase. The spin structure in YFO has a net ferromagnetic moment in the *z* direction, $<S^{z}>$. The DMI, which is perpendicular to the easy axis, causes an additional canting of the antiferromagnetically ordered spins and creates weak magnetization. The magnetic field is applied in the *z* direction.

From the spin Green’s function $g_{ij}\left(E\right)=\langle \langle S_{i}^{Fe+};S_{j}^{Fe-}\rangle \rangle$ the magnetization $M=\langle S^{z}\rangle$ for arbitrary spin value *S* is calculated as:

(3) $$M\left(T\right)=\frac{1}{N}\sum_{i}\left[\left(S+0.5\right)\coth\left[\left(S+0.5\right)\beta E_{mi}\right)]-0.5\coth(0.5\beta E_{mi}\right],$$

where $\beta =1/k_{B}T$, $k_{B}$ is the Boltzmann constant and *T* is the absolute temperature. $E_{mi}$ is the spin excitation energy. *J* is renormalized through the spin-phonon interactions *F* and *R* as well as the magnetoelectric coupling *g* to $J_{eff}=J_{1}+2F^{2}/\left(\omega_{0}-MR\right)+2gP^{2}\cos^{2}\theta$.

The spin-phonon interaction in YFO observed by Raut et al. [8] and Coutinho et al. [10] is taken into account in order to obtain correct results:

(4) $$H_{sp-ph}=\frac{1}{2}\sum_{i,j,k}F\left(i,j,k\right)Q_{i}S_{j}^{z}S_{k}^{z}-\frac{1}{4}\sum_{i,j,r,s}R\left(i,j,r,s\right)Q_{i}Q_{j}S_{r}^{z}S_{s}^{z}+h.c.$$

where *F* and *R* are the spin-phonon coupling constants in the first and second order.

The anharmonic phonon-phonon interactions are given by:

(5) $$\begin{array}{ccc} H_{ph} & = & \frac{1}{2!}\sum_{i}\omega_{0i}a_{i}a_{i}^{+}+\frac{1}{3!}\sum_{i,j,r}B\left(i,j,r\right)Q_{i}Q_{j}Q_{r}\\ & + & \frac{1}{4!}\sum_{i,j,r,s}A\left(i,j,r,s\right)Q_{i}Q_{j}Q_{r}Q_{s}, \end{array}$$

where $Q_{i}$ and $\omega_{0i}$ are the normal coordinate and frequency of the lattice mode.

From the phonon Green’s function, defined via the phonon creation $a^{+}$ and annihilation *a* operators

(6) $$G_{ij}\left(t\right)=\langle \langle a_{i}\left(t\right);a_{j}^{+}\rangle \rangle$$

is observed the phonon energy ω and phonon damping γ

(7) $$\gamma =\gamma_{sp-ph}+\gamma_{ph-ph}$$

using the full Hamiltonian and the method of Tserkovnikov [31].

The Ising model in a transverse field describes the ferroelectric properties. It can be applied to order-disorder (KH${}_{2}$PO${}_{4}$) and displacive (BaTiO${}_{3}$) type ferroelectrics [32,33]. The Hamiltonian reads:

(8) $$H_{e}=\Omega \sum_{i}B_{i}^{x}-\frac{1}{2}\sum_{ij}\left(1-x^{\prime }\right){\tilde{J}}_{ij}B_{i}^{z}B_{j}^{z},$$

where $B_{i}^{x}$, $B_{i}^{z}$ are the spin-1/2 operators of the pseudo-spins, ${\tilde{J}}_{ij}$ denotes the pseudo-spin interaction, Ω is the tunneling frequency, and $x^{\prime }$ is the concentration of the doped ions at Y states.

The Y ion displacement and the FeO${}_{6}$ octahedral distortion cause the spontaneous polarization [34,35], which is calculated to be:

(9) $$P_{s}=\left[\frac{1}{N^{\prime }}\sum_{i}\langle B_{i}^{x}\rangle ;0;\frac{1}{N^{\prime }}\sum_{i}\langle B_{i}^{z}\rangle \right].$$

$H_{me}$ defines the magnetoelectric interaction between the two subsystems:

(10) $$H_{me}=-\lambda \sum_{ij}\left(P_{s}\times e_{ij}\right)\cdot \left(S_{i}\times S_{j}\right).$$

where λ is the coupling constant and $e_{ij}$ is the unit vector along the direction between the nearest-neighbours Fe${}^{3+}$-ions.

The band gap energy $E_{g}$ of YFO is defined by the difference between the valence and conduction bands:

(11) $$E_{g}=\omega^{+}\left(k=0\right)-\omega^{-}\left(k=k_{\sigma }\right).$$

The electronic energies

(12) $$\omega^{\pm }\left(k\right)=\epsilon_{k}-\frac{\sigma }{2}I\langle S^{z}\rangle$$

are observed from the Green’s function $g\left(k,\sigma \right)=\ll c_{k,\sigma };c_{k\sigma }^{+}\gg$, σ=±1, $c_{i\sigma }^{+}$ and $c_{i\sigma }$ are Fermi operators, and *I* is the s-d interaction constant [36].

## 3. Results and Discussion

A certain Fe-spin is fixed in the center of the nanoparticle with an icosahedral symmetry. All spins are included into shells numbered by n=1,...,N. n=1 denotes the central spin and n=N represents the surface shell [37].

The numerical calculations are made using the following model parameters: *J* = −13.8 cm${}^{-1}$, $J^{\prime }$ = −3.45 cm${}^{-1}$, $\tilde{J}$ = 575 cm${}^{-1}$, Ω = 21.4 cm${}^{-1}$, *D* = 4.25 cm${}^{-1}$, *K* = 0.09 cm${}^{-1}$, λ = 1.4 cm${}^{-1}$, $T_{N}$ = 640 K, $T_{C}$ = 420 K [2,38], *F* = 21 cm${}^{-1}$, *R* = −18 cm${}^{-1}$, *B* = − 3 cm${}^{-1}$, and *A* = 6.6 cm${}^{-1}$.

### 3.1. Size and Shape Dependence of the Magnetization

**Figure 2:**
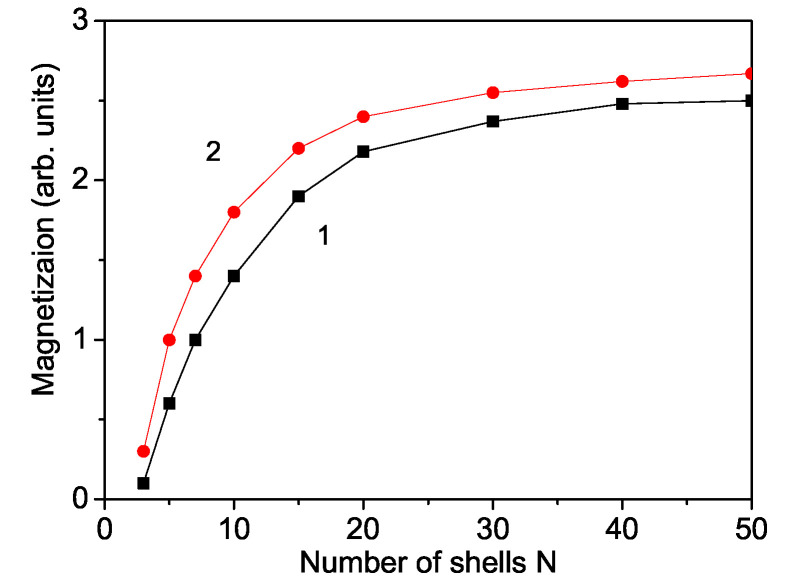
(Color online) The magnetization $M_{s}$ as a function of size and shape in a YFO nanoparticle for *T* = 300 K, $J_{s}=0.8J_{b}$, *h* = 100 Oe. (1) Spherical and (2) cylindrical.

We will first demonstrate the size effects on the saturation magnetization $M_{s}$ in a YFO nanoparticle. It must be noted that a weak magnetization in the case of antiferromagnetic nanoparticles can be due to uncompensated spins at the surface [39]. The exchange interaction constants on the surface, $J_{s}$, can be different than the bulk interaction constants, $J_{b}$, due to the reduced symmetry on the surface. We take for the numerical calculations the relation $J_{s}<J_{b}$. It must be noted that there is a competition between weak-ferromagnetic and antiferromagnetic interactions, which leads to the magnetic properties of a YFO nanoparticle. The results can be seen in Figure 2. The magnetization $M_{s}$ decreases with decreasing nanoparticle size in concordance with the experimental data of Sui et al. [40] and Popkov et al. [41]. This reduction could be due to the existence of a spin-disordered surface layer, in which the thickness is larger than that of the lattice parameters in YFO. The investigations suggest a critical size of around $N_{cr}$ = 3 shells, i.e., ∼6 nm, below which there cannot exist a magnetic phase. Below $N_{cr}$, we have superparamagnetism. Let us emphasize that, by the numerical calculations, we can enhance the shells and for about N = 50 shells, i.e., about 100 nm (see Figure 2), in principle we reach the limit of the nanoparticle size which depends on the model parameters.

The magnetic properties of YFO nanoparticles are shape dependent; see Figure 2, curve 2. The cylindrical nanoparticles show a higher saturation magnetization $M_{s}$ than the spherical ones. A similar result has been observed by Yuan at al. [42]. A strong dimensional influence on the magnetic properties of YFO nanopowders was observed also by Popkov et al. [41].

### 3.2. Electric Field Dependence of the Polarization

**Figure 3:**
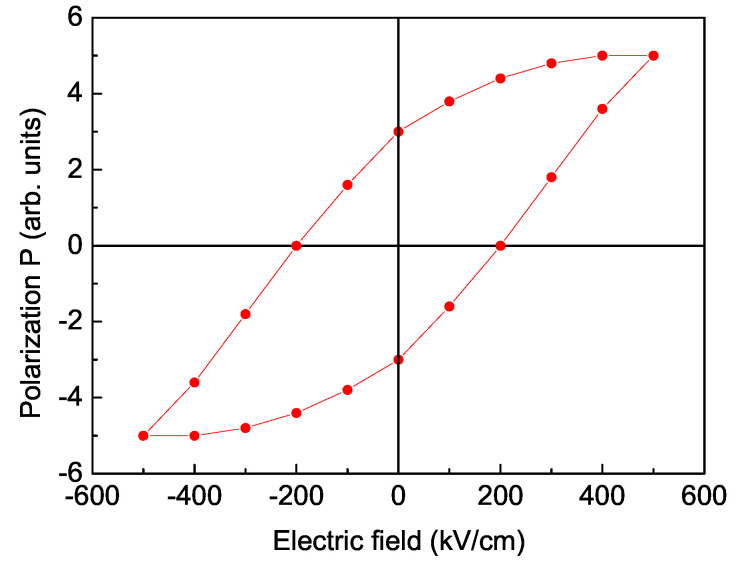
Electric field dependence of the spontaneous polarization $P_{s}$ of a YFO nanoparticle for *T* = 300 K, $J_{s}^{\prime }=0.8J_{b}^{\prime }$ and *N* = 10 shells.

In order to show the multiferroic properties of YFO, we have calculated the polarization of a YFO nanoparticle with *N* = 10 shells. The result is presented in Figure 3. The observed polarization loop provides evidence for the ferroelectric character and supports the multiferroism of YFO. A saturation polarization loop at room temperature in YFO nanoparticles is observed experimentally in Ref. [7]. It must be noted that the polarization decreases with decreasing nanoparticle size *N* (not shown here). There are not reported experimental data for P(N).

### 3.3. Ion Doping Effects on the Magnetization

Let us emphasize that the concentration dependence of the magnetization is considered in the interval 0≤x≤0.3, because in the most cases for larger *x* values a secondary phase is still presented.

#### 3.3.1. Co Substitution at the Fe Site

**Figure 4:**
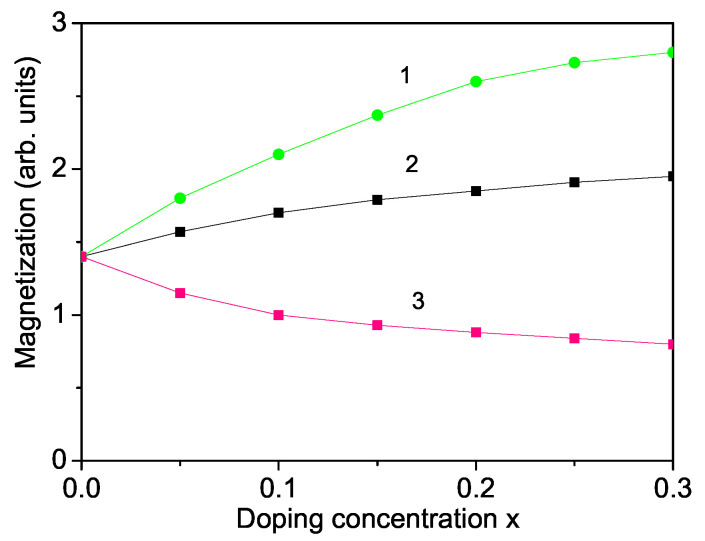
(Color online) The spontaneous magnetization $M_{s}$ as a function of the doping concentration of a (1) Co-doped (with $J_{d}=1.4J_{b}$), (2) Er-doped (with $J_{d}=1.2J_{b}$) and (3) Ti-doped (with $J_{d}=0.8J_{b}$) YFO nanoparticle for *T* = 300 K and *N* = 10 shells.

The magnetic properties of ferrites can be improved through substitution of magnetic or nonmagnetic ions. As a next step, we will study the changes of the magnetic behaviour via doping of different ions at both sites, the Y-cation or Fe-cation site, in YFO nanoparticles. The doping ions substitute the host ions in a given shell, then in the next and so on, shell after shell. They are distributed in shells. By doping with the magnetic Co ion at the Fe site, YFe${}_{1-x}$Co${}_{x}$O${}_{3}$, where the substitution of the Fe${}^{3+}$ ion (*r* = 0.65 $\dot{A}$) by the smaller Co${}^{3+}$ ion (*r* = 0.55 $\dot{A}$) leads to a lattice distortion and to a reduction of the unit cell parameters [12], i.e., to a compressive strain. In our model this means that the exchange interaction parameters between the Fe ions in the defect states $J_{d}$ are larger compared to the undoped values $J_{b}$, $J_{d}>J_{b}$, because *J* is indirectly proportional to the distance between the spins. Moreover, by the calculations, the additive exchange interactions between the Co and the Co-Fe ions must be taken into account. Therefore, the saturation magnetization $M_{s}$ increases with increasing values of the Co ion doping concentration *x*. This can also be explained by the increase of the magnetocrystalline anisotropy, because of the substitution of Co into the sites of Fe. Moreover, the Co${}^{2+}$ doping leads to modification of the Fe-O-Fe angles and to a small quantity of Fe${}^{4+}$ ions appearing to compensate the charge. The observed behavior is in good qualitative coincidence with the data of [12,24,43]. Our results are demonstrated in Figure 4, curve 1. By substituting a Fe ion with a larger Ni${}^{2+}$ ion, one study Nguyen et al. [44] has recently obtained a reduction of the lattice parameters, i.e., a compressive strain. In our model, we take the relation $J_{d}>J_{b}$ and observe again a larger magnetization $M_{s}$ with rising Ni dopants. This is also shown in [44].

#### 3.3.2. Er Substitution at the Y Site

By ion substituting at the Y site in YFO, it is possible to vary the magnetic properties. An increase in $M_{s}$ is also obtained by a substitution of the Y (*r* = 0.90 $\dot{A}$) with the smaller Er ion (*r* = 89 $\dot{A}$), Y${}_{1-x}$Er${}_{x}$FeO${}_{3}$. We have, again, a compressive strain which leads to an increase of $J_{d}$ between the Fe ions, in comparison to the undoped case $J_{b}$, where $J_{d}>J_{b}$. The Fe-O-Fe superexchange interaction can affect the magnetization of Er-doped YFO [13]. This leads to an enhancing of $M_{s}$ (see Figure 4, curve 2), which is in a good qualitative concordance with the results of Cheng et al. [11]. Let us emphasize that the observed saturation magnetization $M_{s}$ is smaller than that in the case of Co ion doping at the Fe site. These enhanced magnetic properties could be of potential use for different applications.

#### 3.3.3. Ca Substitution at the Y Site

We obtain also a small enhancement of the magnetization $M_{s}$ with an increase in the doubly charged ion Ca${}^{2+}$ concentration in YFO at the Y site. The lattice parameters decrease slightly due to the different radii of the Ca (*r* = 1.03 $\dot{A}$) and Y (*r* = 1.06 $\dot{A}$) ions, which leads by using the relation $J_{d}>J_{b}$ to a higher magnetization in the doped YFO compared to the undoped one, in concordance with the results of Tien et al. [45]. The doping with Ca enhances the magnetocrystalline anisotropy of the Y${}_{1-x}$Ca${}_{x}$FeO${}_{3}$ nanoparticles. Moreover, it requires a charge compensation, which can be reached by the transformation of a small part of ions from Fe${}^{3+}$ to Fe${}^{4+}$, changing the Fe-O-Fe angles. With an increase in the Ca concentration, the charge compensating mechanism shifts from electron holes to oxygen vacancies.

#### 3.3.4. Ti Substitution at the Fe Site

Let us emphasize that our model can also explain the experimentally observed reduction of the magnetization $M_{s}$ and the Neel temperature $T_{N}$ in Ti-doped YFO, YFe${}_{1-x}$Ti${}_{x}$O${}_{3}$, nanoparticles [14,15]. This is due to the larger radius of the octahedral Ti${}^{4+}$ ion (*r* = 0.745 $\dot{A}$) relative to that of the host Fe${}^{3+}$ ion (*r* = 0.69 $\dot{A}$), which leads to a tensile strain. Moreover, a change in the valence states of Fe and Ti cations also explains the increase of the volume cell, which leads to the relation $J_{d}<J_{b}$. The result of the magnetization $M_{s}$ as a function of the ion doping concentration *x* is presented in Figure 4, curve 3. Khalifa et al. [14] and Solorzano et al. [15] have reported that doping with Ti${}^{4+}$ ions lowers the Neel temperature $T_{N}$ of YFO nanoparticles. Let us emphasize that our result does not agree with the reported improved magnetic properties of Ti-doped YFO ceramics by Madolappa et al. [16].

#### 3.3.5. Mn Substitution at the Fe Site

We observe also a decrease of the magnetization $M_{s}$ and the Neel temperature $T_{N}$ by doping of YFO with the large anisotropic Mn ion at the Fe site of a YFe${}_{1-x}$Mn${}_{x}$O${}_{3}$ nanoparticle, which is due to the weakening of the superexchange interaction after the Mn${}^{3+}$ substitution. Moreover, there appears to be a spin re-orientation transition and a significant magnetic anisotropy by Mn doping. A similar decrease in the Neel temperature $T_{N}$ and the spontaneous magnetization $M_{s}$ with increasing Mn dopant concentration is observed experimentally by Deka et al. [46,47] and Sundarayya et al. [19].

### 3.4. Ion Doping Effects on the Polarization

**Figure 5:**
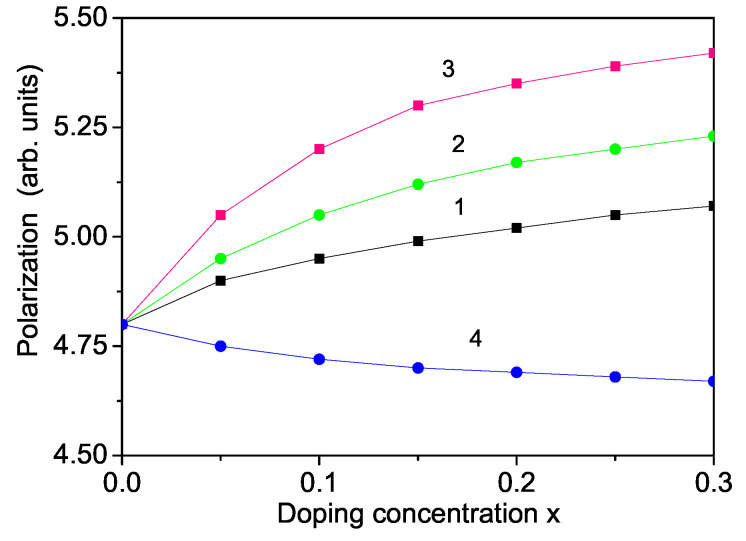
(Color online) The spontaneous polarization $P_{s}$ as a function of the doping concentration of a (1) Mn-doped (with $J_{d}^{\prime }=1.5J_{b}^{\prime }$), (2) Co-doped (with $J_{d}^{\prime }=1.4J_{b}^{\prime }$), (3) Tb-doped (with $J_{d}^{\prime }=1.1J_{b}^{\prime }$) and (4) Sm-doped ($J_{d}^{\prime }=0.6J_{b}^{\prime }$) YFO nanoparticle for *T* = 300 K and *N* = 10 shells.

By doping a YFO nanoparticle with ions at the Y site, we observe an increase in the polarization *P* with increasing doping concentrations of Mn, Co and Yb; in these instances, the ionic radius is smaller than that of the Y ion, i.e., there appears to be a decreasing of the lattice parameters and a compressive strain (see Figure 5, curves 1–3). Conversely, for Sm doping, in which ionic radius is larger that that of Y, we have a tensile strain and *P* decreases with increasing Sm concentration (see Figure 5, curve 4). Unfortunately, there are not many experimental data for P(x). Recently, Martinez et al. [30] and Gonzales [17] have determined the magnetic and ferroelectric properties of Bi${}^{3+}$-doped YFO and observed an enhanced multiferroism. Deka et al. [46] reported an increase in the dielectric constant in Mn-doped YFO.

### 3.5. Temperature and Magnetic Field Dependence of the Phonon Energy

**Figure 6:**
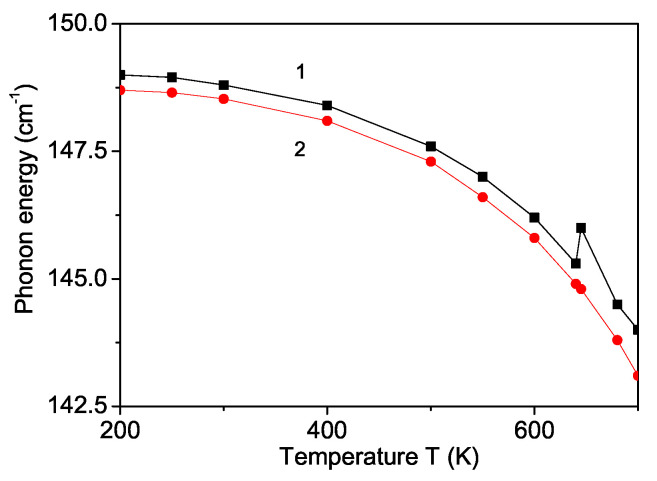
(Color online) Temperature dependence of the phonon mode ω = 149 cm${}^{-1}$ in a YFO nanoparticle with *N* = 10 shells and different magnetic fields *h*: 0 (1); 50 kOe (2).

As a next step, we have investigated the phonon energy ω and phonon damping γ of the $A_{g}$ mode ω = 149 cm${}^{-1}$ [8] in a YFO nanoparticle as a function of temperature with R<0 (see Figure 6, curve 1). It can be seen that at the Neel temperature $T_{N}$, the phonon energy ω (curve 1) shows an anomaly for the case without a magnetic field, *h* = 0, in agreement with Ref. [8]. We obtain a decrease of the phonon mode with increasing temperatures for R<0. This result is due to the strong spin-phonon interaction in YFO [8,9,10]. By applying an external magnetic field, *h* = 50 kOe, ω decreases and the anomaly disappears (Figure 6, curve 2).

### 3.6. Gd and Sm Doping Dependence of the Phonon Energy

We have calculated the effects of ion doping of YFO. For example, by Gd${}^{3+}$ or Sm${}^{3+}$ doping at the Y${}^{3+}$ site, the lattice parameters increase [21,24], respectively, with the increase in Gd${}^{3+}$ or Sm${}^{3+}$ content due to the resulting structure distortion, as the Gd or Sm ionic radius is slightly larger that that of Y, i.e., there is a tensile strain. This strain leads to the relation $J_{d}<J_{b}$ and, through the spin-phonon interaction, influences the phonon properties. The phonon energy decreases with increasing Gd or Sm ion concentrations, in concordance with the results reported by Bharadwaj et al. [21] and Wang et al. [24] for orthorhombic YFO.

It must be noted that Raut et al. [8] have shown that in YFO, both strong electron-phonon and strong spin-phonon coupling exist below the Neel temperature, $T_{N}$, which are also bounded together through spins. The influence of the electron-phonon interaction will be taken into account in a future paper.

### 3.7. Temperature and Magnetic Field Dependence of the Phonon Damping

**Figure 7:**
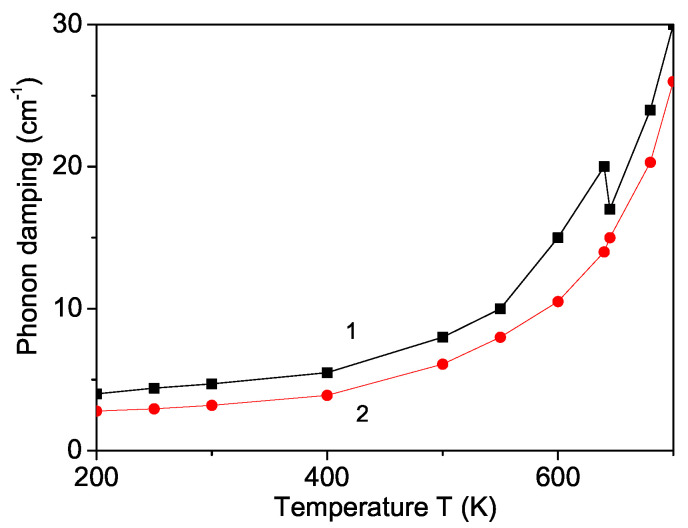
(Color online) Temperature dependence of the damping of the phonon mode ω = 149 cm${}^{-1}$ in a YFO nanoparticle with *N* = 10 shells and different magnetic fields *h*: 0 (1); 50 kOe (2).

The temperature dependence of the phonon damping γ is also calculated. γ enhances with increasing temperature (see Figure 7, curve 1) and also shows an anomaly around the Neel temperature, $T_{N}$, which disappears by applying an external magnetic field (see Figure 7, curve 2). Unfortunately, there does not appear to be published experimental data for ω(h) and γ(h) in YFO.

We obtain that by doping with different ions, the phonon damping increases, because it is proportional to $R^{2}$, i.e., the Raman lines are broader [24].

### 3.8. Ion Doping Effects on the Band Gap Energy

#### 3.8.1. Ti Ion Doping at the Fe Site

**Figure 8:**
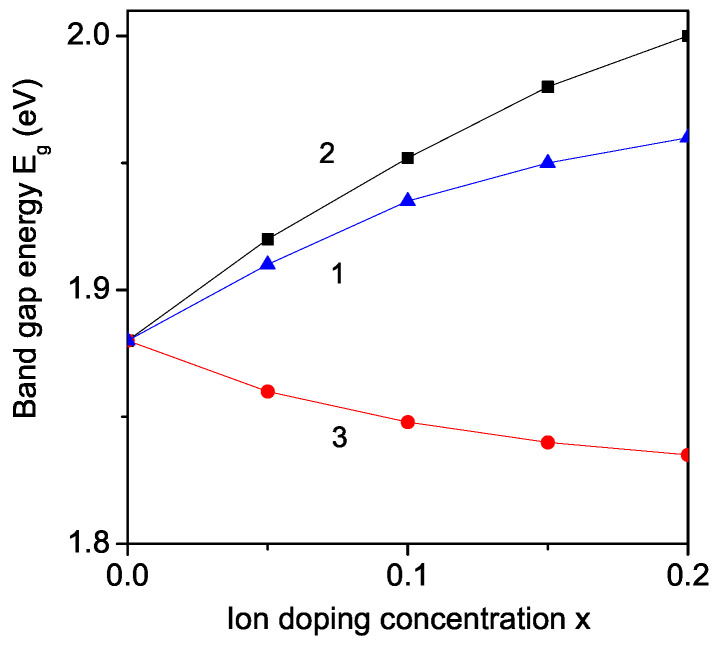
(Color online) Ion doping concentration dependence of the band gap energy Eg of a YFO nanoparticle (N = 10 shells) by (1) Ti doping with $J_{d}=0.8J_{b}$; (2) Sm doping with $J_{d}=0.6J_{b}$; (3) Co doping with $J_{d}=1.4J_{b}$.

The band gap energy $E_{g}$ is observed from Equation (11) for pure and ion-doped YFO nanoparticles. We consider at first the case of a Ti${}^{3+}$-doped YFO nanoparticle, YFe${}_{1-x}$Ti${}_{x}$O${}_{3}$. The lattice parameters increase with increasing Ti dopants because the ionic radius of the Ti ion (*r* = 0.745 $\dot{A}$) is larger compared to the Fe ion (*r* = 0.69 $\dot{A}$). There is a tensile strain, and we use the relation $J_{d}<J_{b}$. We observe an increase in $E_{g}$ (see Figure 8, curve 1).

#### 3.8.2. Sm Ion Doping at the Y Site

A similar enhanced $E_{g}$ is also obtained by doping with Sm${}^{3+}$ (*r* = 1.24 $\dot{A}$) ions at the Y${}^{3+}$ (*r* = 1.06 $\dot{A}$), which also causes a tensile strain and enhanced band gap energy $E_{g}$ (see Figure 8, curve 2), as reported by Bharadwaj et al. [21].

#### 3.8.3. Co Ion Doping at the Fe Site

Otherwise, by Co ion doping, YFe${}_{1-x}$Co${}_{x}$O${}_{3}$, the contrary result is observed—a reduction of the band gap energy $E_{g}$ (see Figure 8, curve 3), in agreement with the results of Wang et al. [24]. This is because the ionic radius of the Co ion (*r* = 0.61 $\dot{A}$) is smaller than that of the Fe ion (*r* = 0.69 $\dot{A}$), which leads to a decrease in the lattice parameters ($J_{d}>J_{b}$) and to a decrease in the band gap energy $E_{g}$.

## 4. Conclusions

In conclusion, we have observed that the spontaneous magnetization $M_{s}$ in a YFO nanoparticle decreases with decreasing particle size and is higher for cylindrical particles than for spherical ones. $M_{s}$ is changed by ion doping, which causes different strains. Moreover, we have discussed substitution at both the Y or Fe sites. Therefore, one can obtain a material with controlled parameters. $M_{s}$ increases with Co or Ni (at the Fe site) and Er (at the Y site) ion doping and decreases with Ti doping (at the Fe site). This significant enhancement in the magnetization is accompanied by a transition from antiferromagnetic to ferromagnetic behaviour, which could be used for various applications. We have tried to clarify the discrepancies of Ti-doped YFO. It must be noted that our results agree with those of Khalifa et al. [14] and Solorzano et al. [15] and disagree with the data of Madolappa et al. [16]. The phonon energy for the $A_{g}$ mode ω = 149 cm${}^{-1}$ and their damping decreases or increases, respectively, with increasing temperature. Both show a kink near $T_{N}$, which disappears by applying an external magnetic field. The band gap energy $E_{g}$ increases with Ti or Sm ion doping and decreases with Co ion doping.

Let us emphasize that they are differences in some properties of hexagonal and orthorhombic YFO NPs which will be investigated in a future paper.

## Data Availability

Derived data supporting the findings of this study are available from the corresponding author upon reasonable request.
